# Supplementary material for: Development in Maillard Reaction and Dehydroalanine Pathway Markers during Storage of UHT Milk Representing Differences in Casein Micelle Size and Sedimentation
Source: Foods. 2022 May 23;11(10):1525. doi: 10.3390/foods11101525 (PMC9140683; doi:10.3390/foods11101525)
Supplement: Supplementary file 1 [file foods-11-01525-s001.zip › foods-1713683-supplementary.pdf]

Supplementary materials

Table S1. Quantity of protein in the sediment during storage.

Day	CN Micelle Size	Protein in sediment (g/L)	
		Undiluted	Diluted
**15**	Small	22.81	0.23
**31**	Small	24.03	0.24
**47**	Small	23.74	0.24
**63**	Small	24.31	0.24
**79**	Small	24.74	0.25
**95**	Small	21.83	0.22
**11**	Large	21.08	0.21
**27**	Large	21.01	0.21
**43**	Large	21.20	0.21
**59**	Large	21.01	0.21
**75**	Large	22.71	0.23
**91**	Large	19.36	0.19
